# NAMPT regulates mitochondria and oxidative stress level for mouse early embryo development

**DOI:** 10.1186/s40659-025-00608-3

**Published:** 2025-05-04

**Authors:** Mei-Hua Liao, Xin Liu, Xiao-Ting Yu, Shun Zhang, You-Zhu Li, Lin-Lin Hu, Shao-Chen Sun, Jun-Li Wang

**Affiliations:** 1 Key Laboratory of Research on Clinical Molecular Diagnosis for High Incidence Diseases in Western Guangxi of Guangxi Higher Education Institutions, Reproductive Medicine of Guangxi Medical and Health Key Discipline Construction Project, Affiliated Hospital of Youjiang Medical University for Nationalities, Baise, China; 2 College of Animal Science and Technology, Nanjing Agricultural University, Nanjing, 210095 China; 3 Department of Reproductive Medical Center, The Affiliated Hospital of Guilin Medical University, Guilin, China; 4 Department of Reproductive Medicine, School of Medicine, The First Affiliated Hospital of Xiamen University, Xiamen University, Xiamen, China

**Keywords:** Oocyte, Actin, Mitochondria, Oxidative stress, Parkinson’s disease

## Abstract

**Background:**

Nicotinamide phosphoribosyltransferase (NAMPT) is an enzyme that involves into NMN-NAD + synthesis which involves into cellular metabolism related with aging, immune function, and neurodegeneration. However, its roles in early embryo development are still unclear.

**Methods:**

In present study we disturbed the NAMPT activity and employed immunofluorescence staining and live cell imaging to explore its roles during early embryo development.

**Results:**

We showed that NAMPT mRNA level was stable during mouse early embryo development, and NAMPT accumulated in the nucleus of blastomeres in mouse embryos. The loss of NAMPT activity disturbed the early cleavage from zygote to 2-cell, 4-cell to morula formation in the dose-dependent manner. We found that NAMPT inhibition disrupted mitochondria function in 2-cell embryos, showing decreased mitochondria number and aberrant accumulation in the blastomeres, which further disturb mitochondrial membrane potential level and elevated ROS level in embryos, indicating the occurrence of oxidative stress. Moreover, NAMPT inhibition also increased the apoptotic index, showing with increased Annexin-V signals and apoptotic gene expression.

**Conclusions:**

Taken together, our study provided the evidence that NAMPT was essential for the mitochondria function to control oxidative stress and apoptosis during mouse early embryo development.

## Introduction

Nicotinamide phosphoribosyltransferase (NAMPT) is an enzyme that plays a crucial role in cellular metabolism, particularly in the synthesis of nicotinamide adenine dinucleotide (NAD+), a vital coenzyme involved in redox reactions, energy production, and cellular signaling. NAMPT is primarily involved in the salvage pathway of NAD + biosynthesis, converting nicotinamide (NAM) into nicotinamide mononucleotide (NMN), which is then converted into NAD+ [1]. NAMPT is expressed in various tissues, and its levels can be influenced by factors like age, stress, inflammation, and metabolic conditions. Because of its central role in NAD + biosynthesis and regulation, NAMPT has been implicated in a range of biological processes, including aging, metabolism, immune function, and neurodegeneration. Modulating NAMPT activity may have therapeutic potential for conditions such as age-related diseases, metabolic disorders, and neurodegenerative diseases like Parkinson’s disease, where NAD + depletion is a contributing factor [2]. Thus, NAMPT is considered an important target for interventions aiming to boost NAD + levels, which could have wide-reaching benefits for cellular health and longevity. The roles of NAMPT in mammalian oocytes were widely reported. It is shown that NAMPT regulates spindle size, which NAMPT deletion causes the acceleration after anaphase-onset and the failure of asymmetry in mouse oocytes [3]. While it causes mitochondria dysfunction and lipid metabolism defects after inhibition in porcine oocytes [4]. Besides, meiotic spindle formation defects are also observed in the porcine oocytes with the inhibition of NAMPT [5]. In the obese mice, NAMPT-induced NAD + decrease caused the decline of oocyte maturation quality [6]. In the aged mice, NAMPT increased and affects mitochondria function through FoxO3a of oocytes [7]. However, the roles of NAMPT during early embryo development is still unclear.

Early embryo development is a highly coordinated process that transforms a fertilized egg from zygote into a multicellular organism capable of implantation into the uterine wall. This process involves a series of cellular divisions and differentiations, leading to the formation of distinct cell layers and tissues. The zygote to blastocyst is key to the establishment of the embryonic body plan and the eventual development of the fetus [8]. Fertilization occurs when a sperm from the male merges with the oocyte from the female, forming a zygote. The zygote is a single diploid cell containing a complete set of chromosomes, half contributed by the sperm and half by the egg. The fertilized egg undergoes a series of rapid cell divisions without an increase in overall size, a process known as cleavage. Cleavage is the first series of cell divisions following fertilization. The zygote undergoes a series of mitotic divisions, producing smaller cells called blastomeres. These divisions occur without growth, so the embryo has no change for the increase in size but rather becomes partitioned into smaller cells. The early stages of cleavage are called 2-cell, 4-cell, 8-cell. After a few rounds of division, the embryo reaches a stage known as the morula, typically around the 16-cell stage. The morula consists of a tightly packed cluster of blastomeres. As cleavage continues, the cells of the morula begin to compact, forming a more organized structure. This process, known as compaction, involves the tightening of cell-cell junctions and the beginning of cellular differentiation. The cells now begin to acquire different identities, and this marks the start of blastocyst formation [9].

Mitochondria play a crucial role in early embryo development by providing the energy needed for cellular processes such as division, differentiation, and growth, and this is medicated by actin filaments [10]. In the zygote and early stages of cleavage, the embryo relies heavily on the maternal supply of mitochondria, which are inherited from the egg [11]. These mitochondria are responsible for generating ATP through oxidative phosphorylation, ensuring that the embryo has the energy required for rapid cell divisions during cleavage. As the embryo transitions from the morula to the blastocyst stage, mitochondria continue to support energy metabolism, but there is also a shift in their function [12]. Mitochondrial ATP production is essential for basic cell functions, while in later stages, mitochondria begin to play an important role in regulating key developmental processes, including calcium homeostasis, where mitochondria help maintain cellular calcium balance which is vital for signaling pathways that control cell fate and differentiation; mitochondria also control metabolism and biosynthesis, since mitochondria support metabolic processes that fuel biosynthesis, such as nucleotide and lipid production, which are essential for tissue formation and growth [13]. Besides, mitochondria are critical for reactive oxygen species (ROS) regulation, where mitochondria generate ROS as byproducts of oxidative metabolism, which is involved in signaling mechanisms that influence cell proliferation and differentiation [14]. This further controls apoptosis which helps eliminate abnormal or damaged cells during development. Thus, mitochondria not only provide the energy necessary for cellular activities during early development but also help regulate the cellular environment through signaling, apoptosis, and metabolism, ensuring proper embryo growth and development [15].

In present study, we explored the roles of NAMPT during mouse early embryo development. By inhibiting its activity, we found that NAMPT was essential for the embryo cleavage, since NAMPT regulated mitochondria function, which maintained ROS level for the control of apoptosis, and our data revealed the important roles of NAMPT in mouse early embryos.

## Materials and methods

### Antibodies and chemicals

Rabbit anti-NAMPT (Vsifatin) was purchased from Abcam (ab236874, Cambridge, UK); FK866 was purchased from MCE (HY-50876, China); Hoechst 33,342 were purchased from Merge Sigma Aldrich (United States); Mito-tracker kit, MMP detection kit, Annexin-V kit and ROS kit were from Thermo Fisher Scientific. Alexa Fluor 594 goat anti-rabbit antibody were purchased from Invitrogen (Carlsbad, CA, USA). All other chemicals were from Sigma Aldrich unless specifically stated.

## Embryo collection and culture

This research project was proved by Animal Research Committee of Nanjing Agricultural University and followed the ethic guideline of Animal Research Committee of Nanjing Agricultural University, China (SYXK2023-008). We injected 5IU PMSG in female mice with 5–6 weeks old with 48 h, and then 5IU hCG was injected. Then the female mice were immediately mated with male mice after the hCG injection. After 16–18 h later we collected the zygotes, removed the cumulus cells by hyaluronidase treatment in the M2 medium. The zygotes were cultured in KSOM culture medium to 24 h, 48 h, and 72 h to get 2-cell, 4-cell and morula embryos.

## FK866 treatment

FK866 was prepared as a 100 mM stock solution in DMSO for storage and diluted to working concentrations of 30 µM or 50 µM in the KSOM culture medium. The zygotes were continuously cultured with FK866 for different culture time to assess the effects of NAMPT inhibition on embryo development.

## Real time RT-PCR and MtDNA number analysis

Real-time RT-PCR was carried out firstly with RNA extraction using the Dynabead mRNA DIRECT kit (Invitrogen Dynal, Oslo, Norway) according to the manufacturer’s instructions. We reverse-transcribed into cDNA using the kit’s reverse transcription components (Takara, Dalian, China.). The reaction was set up in a 20 µL volume containing Faste Universal SYBR Green Master (ROX) 10 µl, forward primer and reverse primer 0.8 µl, and run-on an Applied Biosystems QuantStudio 5 Real-Time PCR System. Cycling conditions were optimized based on the kit’s recommendations. Data were analyzed using QuantStudio Design and Analysis, and gene expression levels were normalized to the control.

To determine mitochondrial DNA (mtDNA) copy number, we extracted total DNA from embryos using a suitable DNA isolation kit (Qiagen DNeasy Blood & Tissue Kit, 69504). And then we measureed the DNA concentration and perform quantitative PCR (qPCR) to evaluate mtDNA levels by targeting the mitochondrial gene The primers were: mtDNA, F: 5΄-CTC AAC CCT AGC AGA AAC CA-3΄; R: 5΄-TTA GTT GGT CGT ATC GGA ATC G-3΄. *18 S*, F: 5΄- CGC GGT TCT ATT TTG TTG GT-3΄; R: 5΄- AGT CGG CAT CGT TTA TGG TC-3΄. The standard real time RT-PCR was then adopted.

## Fluorescence staining

Fluorescence staining was performed to analyze the relative localization in mouse embryos. The protocol was based on our previous studies [16]. For NAMPT staining, samples were fixed with 4% paraformaldehyde for 60 min at room temperature, followed by permeabilization with 0.5% Triton X-100 for 20 min. Blocking was carried out with 1% BSA for 1 h to prevent non-specific binding. Samples were incubated with NAMPT primary antibody overnight at 4 °C, washed with PBS 3 times, and then incubated with secondary antibody for 1 h at room temperature in the dark. DNA were counterstained with Hoechst 33,342 and samples were mounted on the glass slide. Fluorescent signals were visualized and imaged using a a confocal microscope (Carl Zeiss LSM700, Germany). For Mito-tracker, MMP, ROS, Annexin-V staining, we followed the protocol provided by the manufacture.

### Live staining of Mito-tracker

Mito-Tracker staining is the method we used to visualize mitochondria in embryos. To perform the staining, we prepared a working solution of MitoTracker Red CMXRos (Thermo Fisher Scientific, M7512) by diluting it to a final concentration of 50–200 nM in pre-warmed serum-free culture medium. And then we incubated the embryos in this solution at 37 °C for 15 to 30 min in a CO₂ incubator, adjusting the incubation parameters based on sample requirements. After staining, we washed the samples with fresh, warm culture medium to eliminate any unbound dye. For imaging, we transferred the samples to slides with culture medium or PBS and proceed with fluorescence microscopy promptly to maintain signal quality. Live imaging was adopted since the loss of mitochondrial specificity could be occurred after fixation.

For the calculation of abnormal mitochondria distribution, we considered two situations as “Abnormal”: The fluorescence intensity in the blastomeres decreased more than 30%, and there are clustered strong signals (arrows indicated in the images) of mitochondria in the blastomeres.

## NAD + and reactive oxygen species (ROS) detection

For the NAD+/NADH level detection, the commercial kit was adopted (ML092927, Mlbio, China) for the colorimetric assay, and the protocols were followed by the instruction from the manufacturer. To assess the reactive oxygen species (ROS) levels in mouse embryos, CM-H₂DCFDA (Invitrogen, C6827) was used as the fluorescent probe that becomes activated upon oxidation by ROS. We prepared the dye by diluting to a working concentration of 5–10 µM in pre-warmed culture medium without serum. And then we incubate the embryos in the prepared solution at 37 °C in a CO₂ incubator for 15–30 min to facilitate dye uptake and ROS interaction. Following incubation, we washed the embryos thoroughly with fresh, warm medium to eliminate residual dye. ROS levels then was evaluated by measuring fluorescence intensity using a confocal microscope (Carl Zeiss, LSM700) and quantified using Image J software (NIH).

## Fluorescence intensity analysis

To analyze fluorescence intensity, we used Image J software. Specifically, the image file was opened in Image J and was converted to grayscale. The Rectangle or Freehand selection tool was used to outline the region of interest (ROI) where fluorescence was measured. We then analyzed the average fluorescence intensity within the ROI. After selecting the ROI, we obtained the intensity value. The background fluorescence was selected with an area without any sample signal as the control. For multiple ROIs, we repeated the steps for each region, or use ROI Manager to save and analyze multiple regions in batch.

### Statistical analysis

All the experimental groups were repeated at least three biological times. The analysis data were expressed as mean ± standard error. These data were first processed with Excel and then were analyzed and graphed with GraphPad Prism 5 software. Statistical analysis was performed by independent sample t-test to compare whether the difference between the two means was significant. When the P value < 0.05, the difference was considered significant; and P value > 0.1 was considered with no difference.

## Results

### Localization and expression of NAMPT in mouse early embryos

**Fig. 1:**
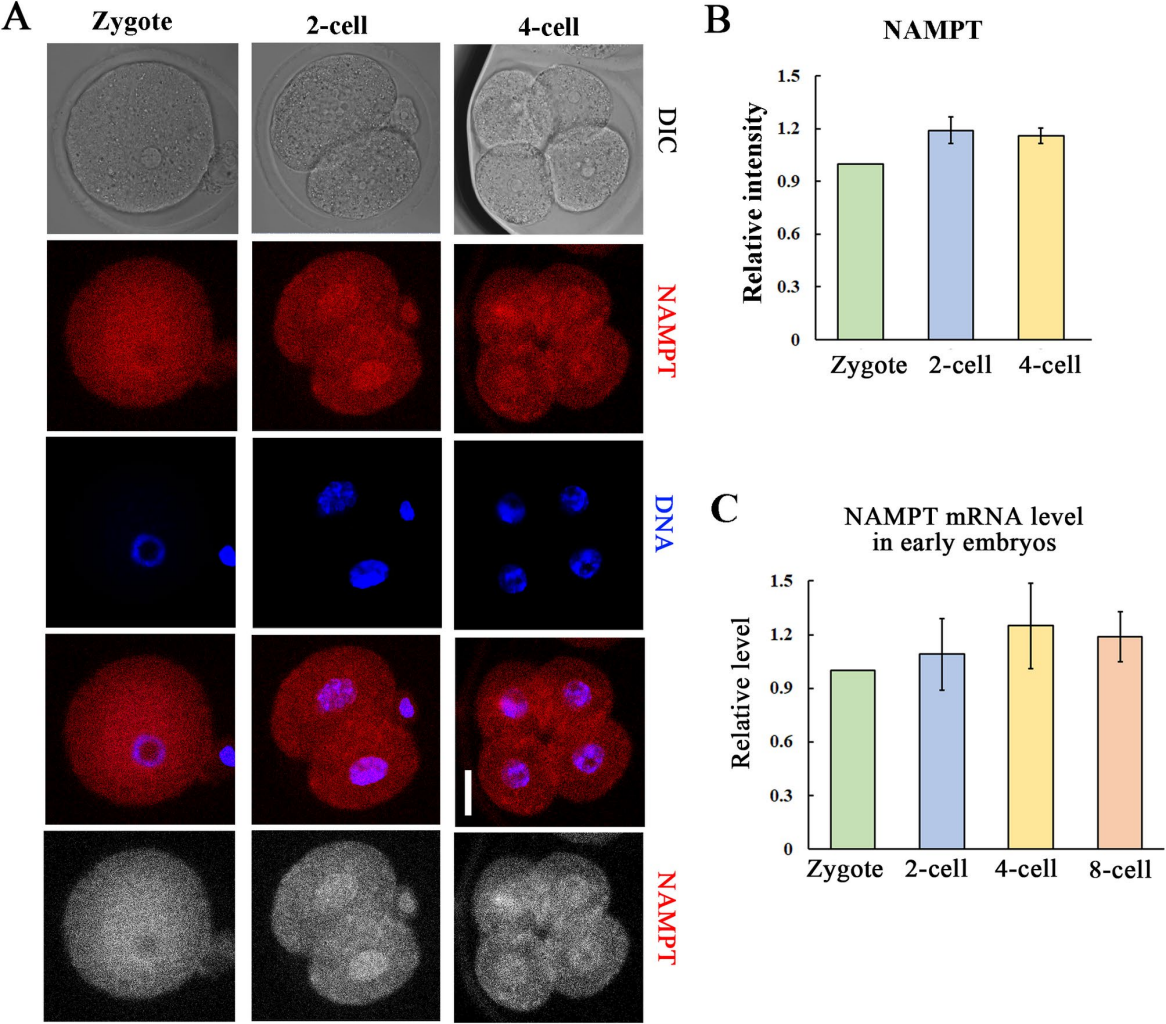
Localization and expression of NAMPT in mouse early embryos. **(A)** NAMPT localization in zygote, 2-cell and 4-cell embryos. NAMPT localized in the nucleus starting from 2-cell embryos. Red, NAMPT; blue, DNA. Bar = 20 μm. **(B)** The relative fluorescence intensity of NAMPT in zygote, 2-cell and 4-cell embryos. **(C)** The relative mRNA level of NAMPT at zygote, 2-cell, 4-cell, 8-cell stage of mouse embryos. No significantly changes were observed between these groups

We first examined the localization and expression of NAMPT in the different stages of mouse early embryos. As shown in Fig. 1A, we found that NAMPT accumulated around the nucleus of zygotes, but localized in the nucleus at 2-cell and 4-cell embryos. The relative fluorescence intensity was 1 vs. 1.19 ± 0.076 vs. 1.16 ± 0.044 (Fig. 1B). We also examined the mRNA expression of NAMPT, and it showed that NAMPT mRNA stably expressed at zygote, 2-cell, 4-cell and 8-cell embryos (1 vs. 1.09 ± 0.2 vs. 1.25 ± 0.24 vs. 1.19 ± 0.14) (Fig. 1C). These data suggested that NAMPT existed in mouse embryos.

### NAMPT activity is essential for mouse early embryo development

**Fig. 2:**
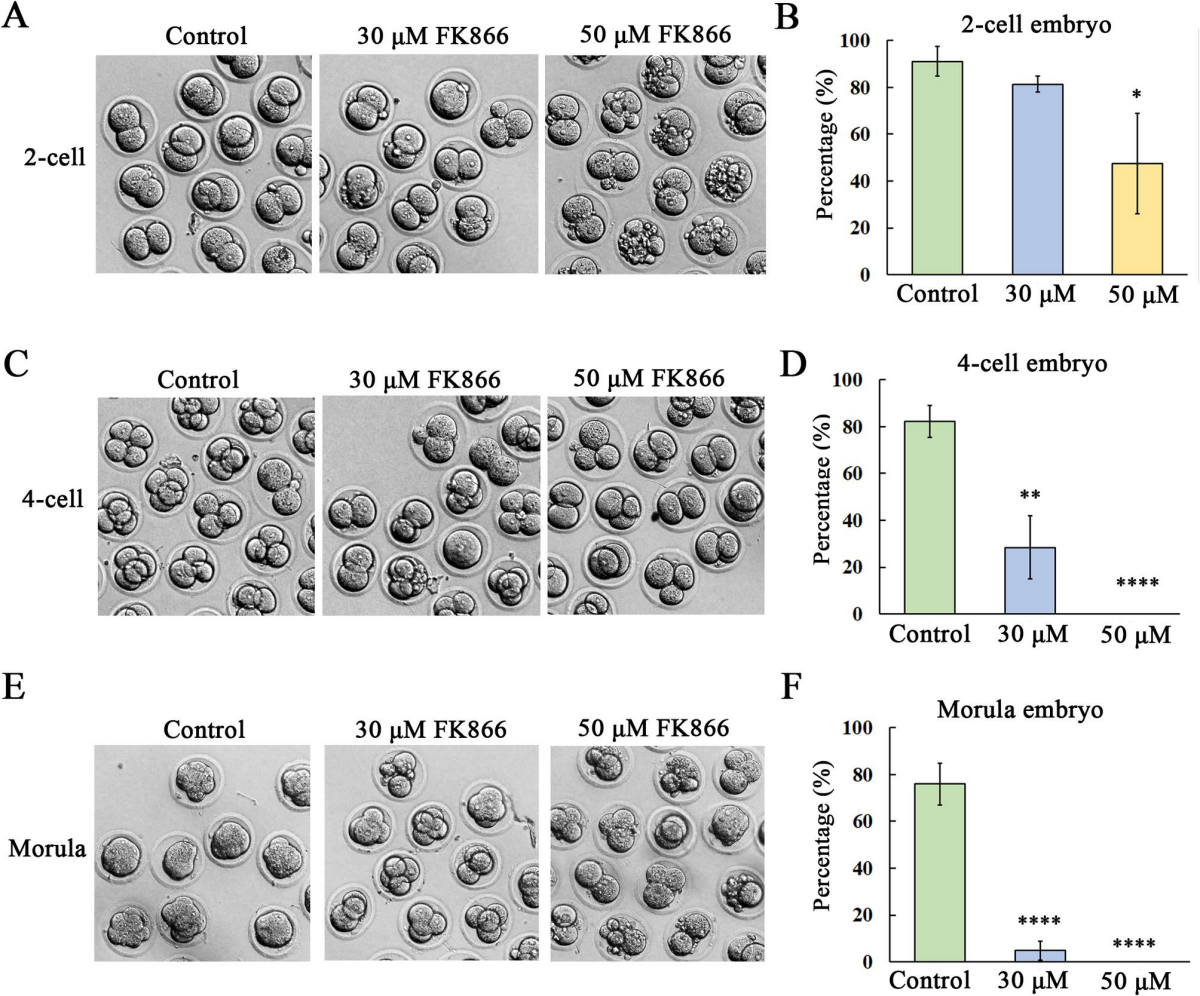
Loss of NAMPT activity disturbs mouse early embryo development. **(A)** Typical images for the 2-cell embryo formation after 30 µM and 50 µM dose of FK866 treatment for 24-hour culture. **(B)** The percentage of 2-cell embryo decreased in the 50 µM FK866 treatment group compared with the control. *, *P* < 0.05. **(C)** Typical images for the 4-cell embryo formation after 30 µM and 50 µM dose of FK866 treatment for 48 h culture. **(D)** The percentage of 4-cell embryo decreased in the 30 µM and 50 µM FK866 treatment groups compared with the control. **, *P* < 0.01; ****, *P* < 0.0001. **(E)** Typical images for the morula embryo formation after 30 µM and 50 µM dose of FK866 treatment for 72 h culture. **(F)** The percentage of morula embryo decreased in the 30 µM and 50 µM FK866 treatment groups compared with the control. ****, *P* < 0.0001

To investigate that whether NAMPT has functions in mouse embyos, we used the specific inhibitor of NAMPT FK866, which the doses were based on previous studies [4]. We showed that 30 µM FK866 treatment for 24 h did not affect the first cleavage to 2-cell embryo, however, 50 µM FK866 caused the failure of 2-cell embryo formation. This was confirmed by the statistical data for the rate of 2-cell embryos (Control, 91.14% ± 6.31%; 30 µM group: 81.36% ± 3.38%; 50 µM group: 47.49% ± 21.56%, *P* < 0.05) (Fig. 2B). While the 4-cell embryo formation was severely affected after 48 h culture: There are few embryos developed to 4-cell stage under 30 µM FK866 treatment, while there were no 4-cell embryos in the 50 µM treatment group (Fig. 2C). The rate of 4-cell embryo in the treatment groups was significantly lower than the control group (Control, 82.13% ± 6.84%; 30 µM group: 28.38% ± 13.44%, *P* < 0.01; 50 µM group: 0, *P* < 0.0001) (Fig. 2D). When the embryos were culture to 4 days, most control embryos developed to morula stage, however, there were almost no morula embryos in the 30 µM and 50 µM treatment groups (Fig. 2E), with significant difference for the ratio data (Control, 76.08% ± 8.94%; 30 µM group: 4.74% ± 4.14%; 50 µM group: 0, *P* < 0.0001) (Fig. 2F). These data suggested the necessary roles of NAMPT for mouse early embryo development.

### NAMPT regulates mitochondria distribution during mouse early embryo development

**Fig. 3:**
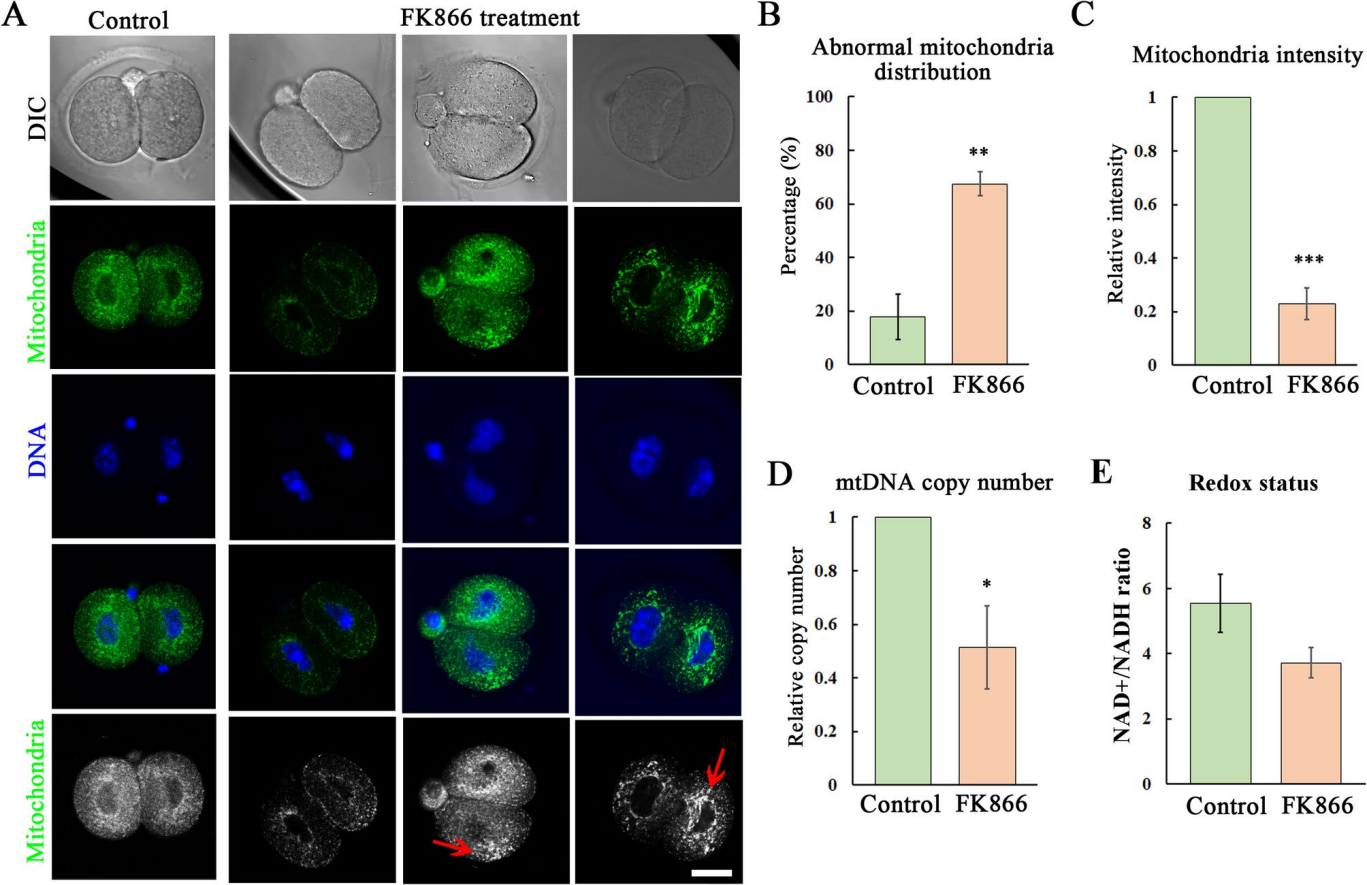
Loss of NAMPT activity disrupts mitochondria distribution in mouse embryos. **(A)** Typical images for the 2-cell embryo staining with Mito-tracker after 50 µM dose of FK866 treatment. Arrows indicated the aberrant distribution of mitochondria. Red, mitochondria; blue, DNA. Bar = 20 μm. **(B)** The percentage of abnormal mitochondria distribution increased in the FK866 treatment groups compared with the control. ***, *P* < 0.01. **(C)** The fluorescence intensity data for the mitochondria showed the significantly decrease after inhibition of NAMPT activity. ***, *P* < 0.001. **(D)** The relative mtDNA number after inhibition of NAMPT activity in the 2-cell embryos. *, *P* < 0.05. **(E)** The NAD+/NADH ratio decreased after inhibition of NAMPT activity. *, *P* < 0.05

We next tried to explore how NAMPT affected embryo development, based on previous studies, we first examined the mitochondria distribution by Mito-tracker staining. As shown in Fig. 3A, the mitochondria were distributed uniformly in the cytoplasm of blastomeres of 2-cell embryos; however, after inhibition of NAMPT activity, there are two phenotypes: (1) the mitochondria showed aberrant accumulation in the cytoplasm, or (2) the fluorescence signals decreased in the treatment groups. We analyzed the ratio of abnormal mitochondria localization, and a significantly increase was observed (Control, 17.8% ± 8.6%; 50 µM group: 67.43% ± 4.5%, *P* < 0.01) (Fig. 3B). Moreover, we also showed the fluorescence intensity data of Mito-tracker remarkably decreased (1 vs. 0.23 ± 0.06, *P* < 0.001, the intensity analysis includes the whole blastomere with the aberrant accumulation) (Fig. 3C). To further confirm this finding, we examined the mitochondria number by mtDNA copy number detection, and similar with the intensity data, the relative mitochondria number was also decreased in the FK866 treatment group (1 vs. 0.51 ± 0.16, *P* < 0.05) (Fig. 3D). To further confirm this, we examined NAD+/NADH ratio, and the data showed that after FK866 treatment, the ratio was decreased (5.54 ± 0.89 vs. 3.72 ± 0.46, *P* < 0.05) (Fig. 3E). These data suggested that the inhibition of NAMPT disrupted mitochondria function in mouse embryos.

### Loss of NAMPT activity induces oxidative stress in mouse embryos

**Fig. 4:**
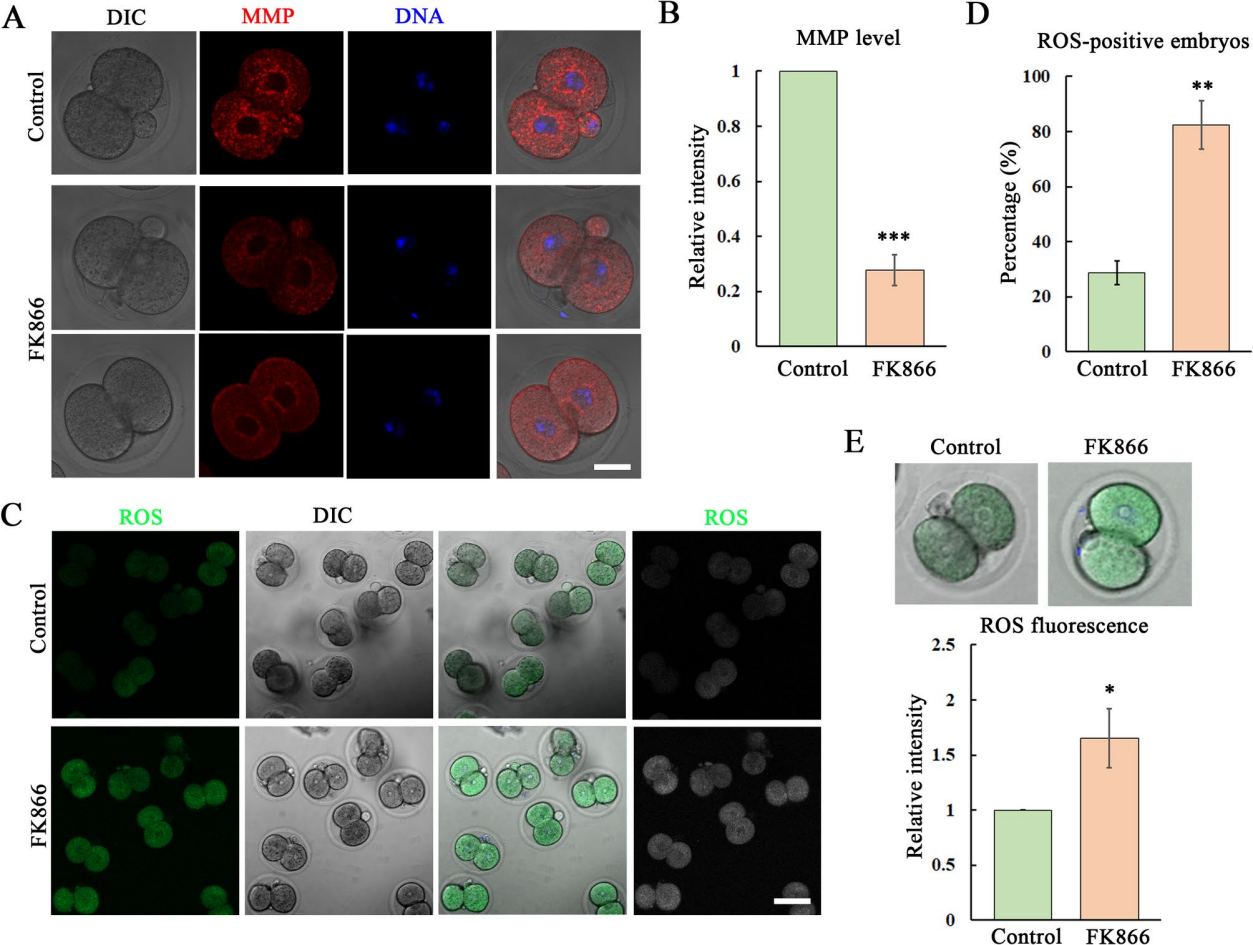
Loss of NAMPT activity induces oxidative stress in mouse embryos. **(A)** Typical images for the 2-cell embryo staining with MMP after 50 µM dose of FK866 treatment. Red, MMP; blue, DNA. Bar = 20 μm. **(B)** The fluorescence intensity data for the MMP showed the significantly decrease after inhibition of NAMPT activity. ***, *P* < 0.001. **(C)** Typical images for the 2-cell embryo staining with ROS after 50 µM dose of FK866 treatment. Green, ROS. Bar = 100 μm. **(D)** The percentage of ROS positive embryos increased in the FK866 treatment groups compared with the control. ***, *P* < 0.01. **(E)** The fluorescence intensity data for the ROS showed the significantly increase after inhibition of NAMPT activity. *, *P* < 0.05

Due to the close relation between mitochondria and oxidative stress, we next examined the mitochondrial membrane potential (MMP) and ROS level, and as shown in Fig. 4A, in the control embryos MMP signals were observed around the nucleus of blastomeres, while the MMP level was decreased by the NAMPT inhibition in 2-cell embryos, which was confirmed by the MMP fluorescence intensity analysis data (1 vs. 0.28 ± 0.06, *P* < 0.001) (Fig. 4B), indicating the dysfunction of mitochondria. We then examined the ROS level, as shown in Fig. 4C, there were barely signals of ROS in the control embryos, while a large proportion of embryos showed positive ROS fluorescence signals after FK866 treatment, indicating the occurrence of oxidative stress. And this was also confirmed by the ratio of ROS-positive embryos (28.6 ± 2.26% vs. 82.47 ± 8.85%, *P* < 0.01) (Fig. 4D). Moreover, we also analyzed ROS fluorescence intensity, and the results were consistent with our findings (1 vs. 1.65 ± 0.27, *P* < 0.05) (Fig. 4E). These data suggested that NAMPT is essential for the control of mitochondria-based oxidative stress in mouse embryos.

### Loss of NAMPT activity induces early apoptosis in mouse embryos

**Fig. 5:**
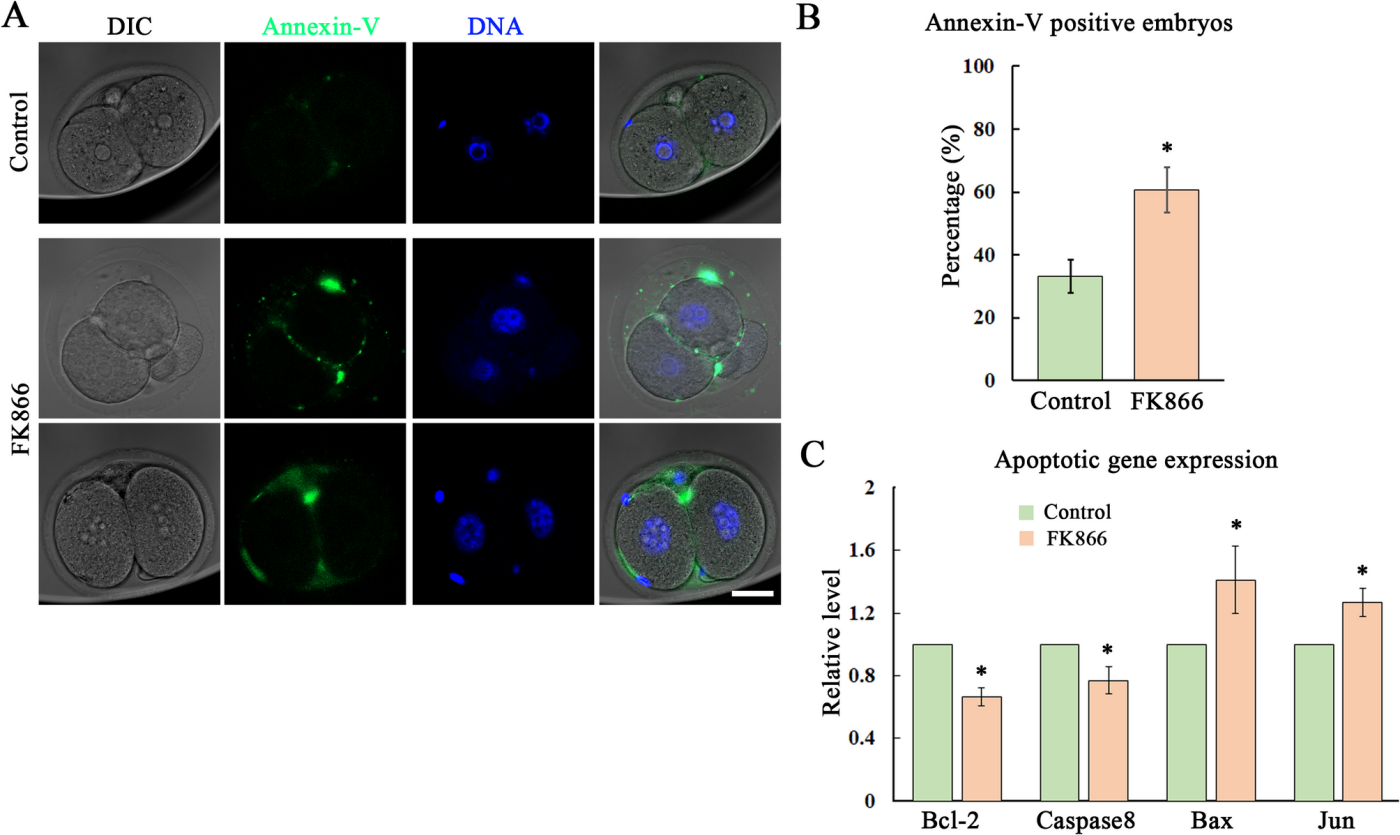
Loss of NAMPT activity induces early apoptosis in mouse embryos. **(A)** Typical images for the 2-cell embryo staining with Annnexin-V after 50 µM dose of FK866 treatment. Green, Annexin-V; blue, DNA. Bar = 20 μm. **(B)** The percentage of Annexin-V positive embryos increased in the FK866 treatment groups compared with the control. *, *P* < 0.05. **(C)** The mRNA level of apoptotic gene after FK866 treatment, which these genes showed altered expression level after inhibition of NAMPT activity. *, *P* < 0.05

Oxidative stress could induce apoptosis, we then examined the apoptotic signals. As shown in Fig. 5A, there are barely signals of Annexin-V, the marker of early apoptosis in the control 2-cell embryos, while there are clearly clustered Annexin-V signals in the treatment embryos, especially at the gap junction of blastomeres. We showed that the rate of Annexin-V positive embryos was significantly higher than the control group after FK866 treatment (33.23 ± 5.3% vs. 60.6 ± 7.18%, *P* < 0.05) (Fig. 5B). Moreover, we also examined the apoptosis-related genes expression, and the real time RT-PCR results showed that the mRNA level of Bcl-2 (1 vs. 0.67 ± 0.06), Caspase 8 (1 vs. 0.77 ± 0.09), Bax (1 vs. 1.41 ± 0.22) and Jun (1 vs. 1.27 ± 0.09) were all altered after the inhibition of NAMPT activity (Fig. 5C), which confirm confirmed the occurrence of apoptosis in mouse early embryos.

## Discussion

As the enzyme for NAD + pathway, NAMPT was reported to involves into multiple cellular events with different cell models, except the early embryos. In present study we reported the important roles of NAMPT in early embryos with mouse model. We showed that NAMPT regulated mitochondria function for the control of ROS level, which prevented the apoptosis from early embryo development.

The localization of NAMPT was widely reported in multiple cell types of reproductive system including trophoblast JEG-3 cells [17], granulosa cells [18], oocytes [4] with several disease model such as ovarian cancer [19] and PCOS [20]. While our data indicated that NAMPT had specific localization pattern from 2-cell embryos, the stage of mouse zygotic genome activation. Moreover, loss of NAMPT activity could disturb embryo development of first cleavage, 4-cell and morula formation due to the dose of inhibitor used, these data implied the essential roles of NAMPT during early embryo development. It should be noted that NAMPT seems not only block zygotic genome activation, instead it affects the general cleavage process of blastomeres since only high dose treatment blocked the 2-cell formation. To explore how NAMPT affect embryo development, we first focused on mitochondria, since as the NAD + metabolism-related molecules, NAMPT was widely reported to associate with mitochondria function. It is shown that NRF2 is the downstream molecule of NAMPT, and NAMPT regulates mitochondria content and membrane potential from NRF2-PPAR-AMPK pathway for the cell survivial [21]. While loss of AMPK activity could disrupt mitochondria-based oxidative stress in oocytes during aging [22]. During skeletal muscle development, NAMPT could maintain Ca2 + homeostasis and mitochondria integrity [23]. Moreover, LaminA/C associates with PGC1 and NAMPT pathway for the mitochondria function, which is related with aging process [24]. Similarly, we showed the aberrant mitochondria distribution, and reduced mitochondria number, and disrupted mitochondria function shown by MMP level after loss of NAMPT activity in mouse 2-cell embryo, indicating that NAMPT ensure mitochondria function for the normal embryo development. While the effects of NAMPT on mitochondria energy production is highly through its regulation on NAD+/NADH redox status. It should be noted that mitochondria distribution was dynamic during cell proliferation, and we only showed that the localization of mitochondria in 2-cell G phase was disturbed, whether its distribution was affected at M phase still needs further study.

Mitochondria are the main source of cellular ROS, which is integral components for multiple cellular processes. The excessive ROS then induces oxidative stress for damaging cells. The nexus of cellular homeostasis between ROS and mitochondria is well understood [25]. NAMPT is essential for NAD metabolism, and the deacetylation of NAMPT could increase NAD synthesis and protect organs from oxidative stress [26]. It is shown that NAMPT could inhibit glucose deprivation-related oxidative stress in breast cancer due to its roles on metabolic stress [27]. While inhibition of NAMPT could reduced oxidative stress, inflammation and keratinocyte DNA damage in zebrafish models of chronic skin inflammation, and this could be reversed by NAD + supplementation [28]. In human THP-1 and HT-22 cells, a recent study revealed that a novel NAMPT positive allosteric modulators (N-PAMs) mitigates elevated ROS in neurons stressed with TNFα and glutamate [29]. While our data also showed that inhibition of NAMPT activity induces evaluated ROS level for oxidative stress in mouse embryo model, which was consistent with previous findings, showing its conserved roles for avoiding oxidant damage.

Oxidative stress is the one main cause for the occurrence of apoptosis and autophagy, although there is mitochondria-dependent and independent pathways [30]. NAMPT is shown to related with apoptosis in several models such as aging, cancer, inflammation and metabolic disorders. Both pharmacological and genetic inhibition of NAMPT induced apoptosis through the activity of the tumor suppressor p53 in human melanoma cells [31]. FK-866-treated cells show increased apoptosis and the expression of anti-apoptotic factors components of the mitochondria-dependent intrinsic apoptotic pathway significantly decreased in MDPC-23 cells [32]. Treatment with another NAMPT inhibitor KPT-9274 suppressed the conversion of saturated fatty acids to monounsaturated fatty acids resulting in apoptosis of AML cells, indicating the potential therapeutic strategy for targeting leukemic stem cells [33]. NAMPT inhibition with STF-118,804 (STF) decreased ATP, induced apoptosis, and reduced NB stem cell neurosphere formation, suggesting its roles on neuroblastoma cell death and tumor growth [34]. In reproductive system, it is shown that NAMPT inhibition affect ovarian proliferation, and induces apoptosis and disturbs steroidogenesis in pre-pubertal mice ovary [35]. We also observed the occurrence of early apoptosis in mouse embryos after inhibition of NAMPT, which may be the results from its effects on mitochondria-dependent oxidative stress.

Taken together, our study provided the evidences for the important roles of NAMPT on mouse early embryo development through its effects on mitochondria function and the control of oxidative stress-related apoptosis.

## Data Availability

The data underlying this article are available in the article and in its online supplementary material.
